# Adenovirus 41 diversity in Arizona (USA) using wastewater-based epidemiology, long-range PCR, and pathogen sequencing between October 2019 and March 2020

**DOI:** 10.1017/S095026882400133X

**Published:** 2024-11-18

**Authors:** Temitope O. C. Faleye, Peter Skidmore, Amir Elyaderani, Sangeet Adhikari, Nicole Kaiser, Abriana Smith, Allan Yanez, Tyler Perleberg, Erin M. Driver, Rolf U. Halden, Arvind Varsani, Matthew Scotch

**Affiliations:** 1 Biodesign Center for Environmental Health Engineering, Biodesign Institute, Arizona State University, Tempe, AZ 85287, USA; 2 College of Health Solutions, Arizona State University, Tempe, AZ, USA; 3 School of Sustainable Engineering and the Built Environment, Arizona State University, Tempe, AZ 85287, USA; 4 OneWaterOneHealth, Nonprofit Project of the Arizona State University Foundation, Tempe, AZ, USA; 5 Biodesign Center for Fundamental and Applied Microbiomics, Center for Evolution and Medicine, School of Life Sciences, Arizona State University, Tempe, AZ 85287, USA

**Keywords:** adenovirus type 41, high-throughput nucleotide sequencing, wastewater-based epidemiological monitoring, Arizona, USA

## Abstract

By coupling long-range polymerase chain reaction, wastewater-based epidemiology, and pathogen sequencing, we show that adenovirus type 41 hexon-sequence lineages, described in children with hepatitis of unknown origin in the United States in 2021, were already circulating within the country in 2019. We also observed other lineages in the wastewater, whose complete genomes have yet to be documented from clinical samples.

## Summary

Adenovirus type 41 (Ad41) is a double-stranded DNA virus with non-enveloped capsid and etiological agent of diarrhoea in children aged below 2 years. It often results in fatal systemic disseminated disease in immunocompromised individuals and has more recently been associated with (possibly as a helper virus of Adeno-associated virus type 2) hepatitis of unknown origin in the same demographic [2, 3, 5–7]. Adenoviruses are members of the genus *Mastadenovirus*, family *Adenoviridae*, which are classified into seven species (*Human mastadenovirus A*-*G*) and contain ~100 types. Mastadenovirus types 40 and 41 are the only members of the species *Human mastadenovirus F* and are transmitted via the faecal-oral route, shed in the faeces of infected persons, and are stable in wastewater (WW) for weeks [1, 5].

Using long-range polymerase chain reaction (PCR), WW-based epidemiology (WBE), and pathogen sequencing, we explored Ad41 diversity in WW by analysing 58 samples collected (over 24 h using time- or flow-weighted automated samplers) from 10 different sites in two municipalities (population: ~700,000) in Maricopa County, Arizona (USA) between October 2019 and March 2020. For each of the 6 months (except March 2020), 10 archived samples per month were recovered from the freezer and thawed overnight. Only eight sites were sampled in March 2020 because two of the locations were not collected for logistic reasons attributed to the onset of the COVID-19 pandemic. Samples were size fractionated, and both filtrate and filter-trapped solids (FTS) were concentrated (~1,000×) using 10,000 Da molecular weight cut-off centrifugal filters (see Supplementary Materials for detailed methods). Hence, for each of the 6 months, we studied two concentrates, one for filtrate and one for FTS.

We subjected all 12 concentrates to nucleic acid extraction using the QIAamp viral RNA Mini Kit following the manufacturer’s instructions and amplified the complete Ad41 genome in eight overlapping ~5 kb fragments via two multiplex PCR assays (Supplementary Tables S1 and S2). We confirmed Ad41 presence using a second-round (nested) PCR assay (targeting the fibre gene), which included the pooled first-round amplicons as a template (Tables S1 and S2). Amplicons from the nested assay were Sanger sequenced and the sequences confirmed that all had Ad41. Their respective first-round amplicons were then sequenced on an Illumina sequencer (see Supplementary Materials).

Illumina sequencing of the first-round amplicons from the 12 concentrates yielded 26,191,460 raw reads. Of these, 13,561,483 (52%) mapped to a reference Ad41 complete genome (MW567966, Table S3) identified in a patient in France in 2018 [4]. Reference-guided assembly showed that one of the eight 5 kb first-round PCR assays (which covers the genomic region containing the penton protein complete coding sequence) failed. Thus, we recovered about 87.5% of the Ad41 genome.

Subsequently, we investigated the variant profile of hexon coding sequences and the two (small and large) fibre protein genes. Variant analysis of the genes showed seven, one, and three unique profiles for the hexon, small fibre, and long fibre genes, respectively (Table 1, Tables S4 and S5). The variability detected in the hexon gene region was primarily within the hypervariable region (HVR) (Table 1).

**Table 1.** Amino acid substitutions in the hexon protein gene hypervariable region (HVR) detected by variant analysis of mapped reads in this study and their presence in Ad41 lineages in GenBank. Codon numbering is relative to the hexon protein gene of MW567966 (L1). Note that L1 has the same sequence as H1. Also, * = Insertion, *! = Insertion and deletion, + = present in all members, (+) = present in all some members. Please note that all variant sites called had >1,000× coverage and amino acid substitutions called as belonging to the same hexon variant have variant frequency greater than 20% and mostly within 6% of each other’s value. Also note that H1 = L1, H5 = L2, H3 is a subset of L3, H7 = L5 + L6, and H4 could be H2 + H3

				Hexon variants in WW							Lineages in GenBank					
Hexon (HVR)	S/N	Substitutions	Codon#	H1	H2	H3	H4	H5	H6	H7	L1	L2	L3	L4	L5	L6
1	1	D – DN*	138							**+**					**+**	**+**
	2	F – L	151		**+**		**+**	**+**		**+**		**+**			**+**	
	3	N – S	155							**+**				**+**		**+**
	4	N – D	157		**+**	**+**	**+**	**+**		**+**		**+**	**+**	**+**	**+**	
	5	N - K	160			**+**	**+**			**+**			**+**	**+**		**+**
2	6	G – E	165							**+**				**+**		**+**
	7	T – A	168		**+**	**+**	**+**	**+**	**+**	**+**		**+**		**+**	**+**	**+**
	8	NQ – K*!	170–171		**+**		**+**	**+**		**+**		**+**	**(+)**	**+**	**+**	
3	9	A – T	197		**+**		**+**	**+**		**+**		**+**			**+**	
	10	Q – D	198		**+**		**+**	**+**		**+**		**+**		**+**	**+**	
4	11	S – N	230		**+**		**+**	**+**		**+**		**+**			**+**	
5	12	P – A	252		**+**		**+**	**+**		**+**		**+**		**+**	**+**	
	13	S – N	253		**+**		**+**	**+**		**+**		**+**		**+**	**+**	
	14	E – V	257		**+**		**+**	**+**		**+**		**+**		**+**	**+**	
	15a	S – A	268		**+**		**+**	**+**		**+**		**+**		**+**		
	15b	S – T	268												**+**	
6	16	D – G	281							**+**				**+**		**+**
	17	I – V	287		**+**		**+**	**+**		**+**		**+**			**+**	
7	18	G – S	411					**+**		**+**		**+**			**+**	
	19	Q – G	412					**+**		**+**		**+**			**+**	
	20	T – N	413					**+**		**+**		**+**			**+**	
	21	D – DN*	419					**+**		**+**		**+**	**(+)**	**+**	**+**	
	22	N – T	420					**+**		**+**		**+**	**(+)**	**+**	**+**	

To understand how the variant profiles of Ad41 found in WW in this study track with those present in variants publicly available in GenBank, we collected the hexon protein gene complete coding sequences of the top 100 variants downloaded from a GenBank search using MW567966 as the query. Precisely, we extracted 59 of the variants from complete genomes, whereas the remaining 41 were not part of complete genomes but had the complete hexon protein coding sequence publicly available in GenBank. Phylogenetic and pairwise similarity analyses of these Ad41 hexon gene sequences showed they clustered into six (L1–L6) phylogenetic lineages (Figure S1). Intra-lineage divergence was less than 0.4% (Figure S1), and each lineage had a unique combination of amino acid substitutions in the HVR (Table 1 and Figure S2).

**Figure 1.**
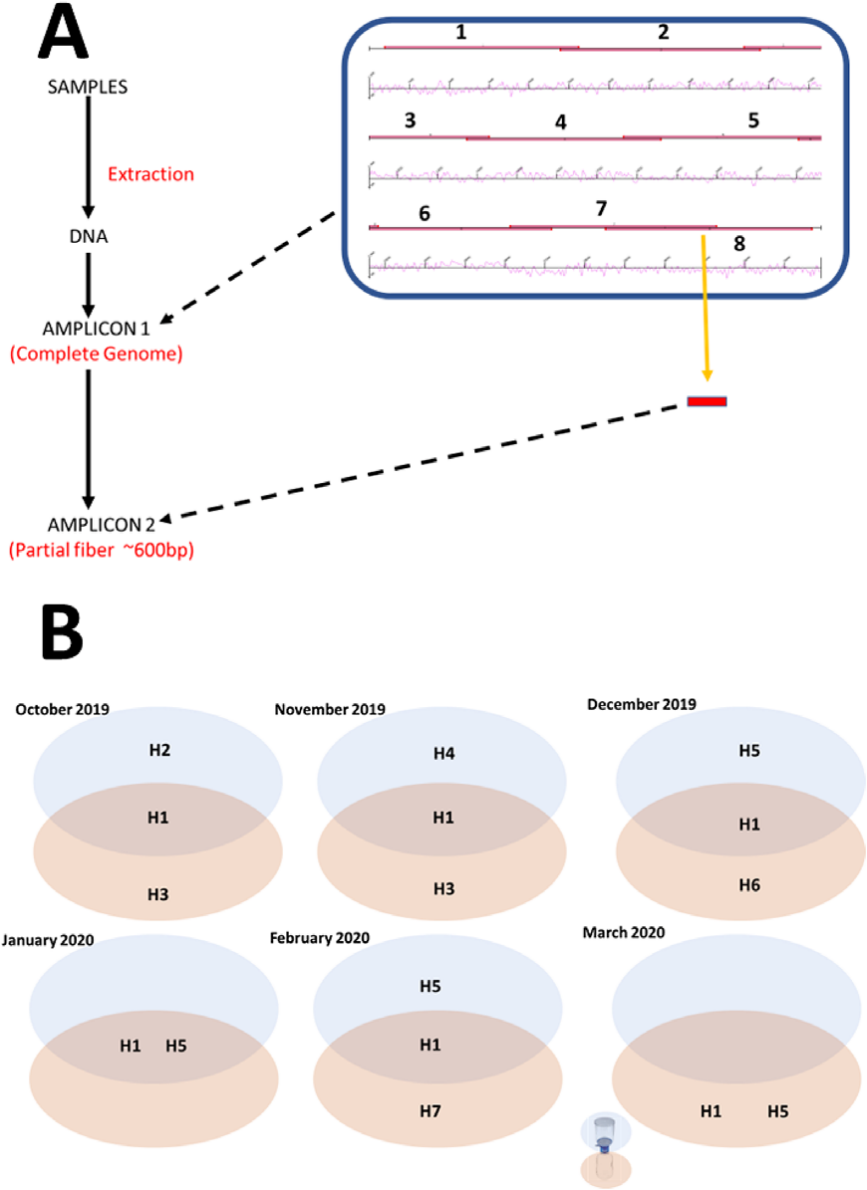
(a) A schematic representation of the workflow described in this study. (b) Hexon protein gene variants identified in different fractions of WW from October 2019 to March 2020. Please note that FTS from March 2020 had Ad41 present based on the second round PCR result. However, only assay 7 (which captures short and long fibre coding region but not hexon) worked in the first-round assay (see Supplementary Table S5). For each month, Ad41 types/variants detected in FTS or filtrate are clustered in blue or beige ovals, respectively. Those detected in both are in the overlapping regions of both ovals.

Comparison of amino acid variation profiles of the hexon genes we found with those in GenBank, showed that hexon variant 1 (H1) = L1, H5 = L2, H3 is a subset of L3, and H7 = L5 + L6 (Table 1). We also found that the amino acid variation profiles H2, H4 (possibly H2 + H3; see Table 1), and H6 were not represented in lineages L1–L6 (Table 1). The seven variant profiles (H1–H7) detected in the concentrates analysed in this study contained between 1 and 22 amino acid substitutions (Table 1). Only in the January 2020, sample did the hexon gene variant profile of the FTS match the filtrate. In the all other months, they were different (Figure 1).

To understand how the Ad41 variants recently identified in children with hepatitis of unknown origin fit into this schema, we aligned the published [2] hexon genes (ON565007–ON565011) with representatives of the six lineages. Our data show that they belong to lineages L1, L2, and L6 with both genomes identified in children with acute liver failure belonging to lineage L6 (Figure S2).

For the long-fibre protein, the F251V substitution was the only amino acid substitution detected in the variant analysis (Table S4 and Figure S3), and when present, was attributed to 38%–55% of mapped reads in all samples. Hence, suggesting that the F251 was equally present in the population. Variant analysis also showed the presence of a variant with a 45 nt (15aa) deletion in the fibre gene in January 2020 (Table S4 and Figure S4). However, it was only found in FTS and not the filtrate (Table S5). For the small fibre gene, the L362F substitution was the only amino acid substitution detected by variant analysis and was present in more than 99% of the raw reads in all samples.

In conclusion, coupling long-range PCR detection with WBGE, we show that all three Ad41 hexon protein lineages recently associated with hepatitis of unknown origin [2] were present in the United States in 2019 (Table 1 and Figure S2). Our data also suggest that there may be circulating Ad41 variants (H2 and H4, Table 1) whose complete genomes have either not been sequenced or sequenced but not publicly available in GenBank. Here, we demonstrate the use of WBGE to monitor Ad41 diversity on a population scale. Specifically, our data show that by targeting a protein-coding region under evolutionary pressure from the immune system (like the hexon protein gene), we can capture diversity (both amino acid substitutions (Table 1) and gross deletions like the 15aa deletion, Figure S4), and we also can use variant profiling coupled with case-based surveillance data to determine which lineages are likely circulating in the population at any point in time (Table 1 and Figure S2).

Our results highlight a limitation of the tiled-amplicon approach for WBGE. Analysis of Ad41 complete genome sequence data publicly available in GenBank (all of which have similarity >98%) showed that recombination is ongoing between the hexon gene and the fibre genes (Figure S3), which are >8 kb apart. However, since the two amplification pools in the tiled amplicon approach are run in different tubes, there is no guarantee that the template genome(s) in both pools are from the same variant; that is, that overlapping tiles are from the same variant and consequently contiguous. Furthermore, short-read sequencing makes it difficult to unambiguously ascertain the co-evolution of distant Ad41 genomic regions (and consequently amino acid substitutions). It might therefore be necessary to implement long-range PCR assays that amplify penton-to-long-fibre or at least hexon-to-long-fibre and couple with long-read sequencing strategies. Such an approach could enable scientists to study the co-evolution of distant Ad41 genomic regions using variants recovered from WW. It might be necessary to also consider this approach for the WBGE of other DNA viruses of clinical significance.

Our data (Figure 1) also showed that commonly performed size fractionation of WW samples prior to ultrafiltration does impact our perception of virus presence and diversity. Hence, prioritizing one sample fraction over the other might result in an incorrect representation of viral diversity. We therefore recommend virus recovery from both partitions (filtrate and FTS) for a more accurate representation of virus presence and diversity in WW samples.

## Supporting information

Faleye et al. supplementary material Faleye et al. supplementary material

## Data Availability

The sequences described in this study have been deposited in SRA under accession numbers SRR21987059 – SRR21987066 and SRR21987070 – SRR21987072.

## Supplementary material

For supplementary material accompanying this paper visit http://doi.org/10.1017/S095026882400133X. click here to view supplementary material
