# Supplementary material for: Adenovirus 41 diversity in Arizona (USA) using wastewater-based epidemiology, long-range PCR, and pathogen sequencing between October 2019 and March 2020
Source: Epidemiol Infect. 2024 Nov 18;152:e142. doi: 10.1017/S095026882400133X (PMC11574596; doi:10.1017/S095026882400133X)
Supplement: Faleye et al. supplementary material [file S095026882400133Xsup001.docx]

**Supplementary File**

**Title:** Adenovirus 41 diversity in Arizona (USA) using wastewater-based epidemiology, long-range PCR, and pathogen sequencing between October 2019 and March 2020

**Authors:** Temitope O.C. Faleye^1^, Peter Skidmore^2^, Amir Elyaderani^2^, Sangeet Adhikari^1,3^, Nicole Kaiser^2^, Abriana Smith^2^, Allan Yanez^1^, Tyler Perleberg^1^, Erin M. Driver^1^, Rolf U. Halden^1,3,4^, Arvind Varsani^5^, Matthew Scotch^1,2^*

1 Biodesign Center for Environmental Health Engineering, Biodesign Institute, Arizona State University, Tempe, AZ 85287, USA.

2 College of Health Solutions, Arizona State University, Tempe, AZ, USA.

3 School of Sustainable Engineering and the Built Environment, Arizona State University, Tempe, AZ, 85287, USA.

4 OneWaterOneHealth, Nonprofit Project of the Arizona State University Foundation, Tempe, AZ, USA.

5 Biodesign Center for Fundamental and Applied Microbiomics, Center for Evolution and Medicine, School of Life Sciences, Arizona State University, Tempe, AZ 85287, USA.

*Correspondence: matthew.scotch@asu.edu

**Methods**

We utilized 58 wastewater samples collected from ten different sites in two municipalities (population: ~700,000) in Maricopa County, Arizona (USA) over a six-month (October 2019 – March 2020) period. For each of the 6 months (except March 2020), we recovered ten archived samples (one per site) per month from the freezer and thawed them overnight. We collected only eight (8) samples in March 2020 because two of the locations were not sampled for logistic reasons due to the onset of the COVID-19 pandemic. We concentrated samples as previously described [1]. Briefly, we filtered 200 ml of wastewater from each of the ten sites using ten 450 nM membrane filters (Thermo Fisher Scientific, Waltham, MA, USA). We pooled and concentrated the filtrates to 2ml (~1000× concentration) using 10,000 Da molecular weight (MW) cutoff centrifugal filters. We also recovered the membrane filters and resuspended the filter-trapped solids (FTSs) by vortexing (Heidolph Instruments, Germany) for 10 minutes at 3000 rpm in a 50 ml centrifuge tube containing 25 ml of sterile PCR grade water with 15 glass beads (3mm, Cole-Parmer, USA). After vortexing, we removed the filters, and centrifuged the mixture for 20 minutes at 3,900 rpm and 4^o^C. We recovered the supernatant, and then pooled and concentrated it to 2 ml using 10,000 Da MW cutoff centrifugal filters. Hence, for each of the 6 months, there were two concentrates, one for filtrate and one for FTS. We subjected all 12 concentrates to nucleic acid extraction using the QIAamp viral RNA MiniKit following the manufacturer’s instructions.

We amplified complete Ad41 genomes in eight overlapping ~5kb fragments in two multiplex assays (Appendix Figure 1 and Appendix Tables 1 and 2). We downloaded Ad41 complete genomes available in GenBank as of 9 May 2022, aligned and submitted to the Primalscheme web server [2] with the amplicon size set to 5,000 bp. We used the designed primers to amplify the eight overlapping amplicons in two multiplex assays. Pool 1 contained assays 1, 3, 5 and 7 while pool 2 contained assays 2, 4, 6 and 8. Please note that assays 7 and 8 both amplify the fiber genes. We pooled the first-round amplicons and used them as a template for a second-round assay targeting the fiber protein gene (Appendix Table 1 and 2). We ran all assays using a BioRad 1000 thermal cycler (BioRad, Hercules, CA, USA). We resolved second-round amplicons on 2% agarose gels stained with GelRed (Biotium, Fremont, CA, USA) and viewed using BioRad Gel Doc XR+ system running Image lab 4.1 software with option to “highlight saturated pixels” enabled (BioRad, Hercules, CA, USA).

We purified the generated amplicons from the second-round assays, subjected them to Sanger sequencing using their respective forward and reverse primers. This was to confirm that the first-round assays worked, and the target virus (and genomic region) was amplified. Subsequently, we purified the first-round amplicons of all samples positive for the second-round assays and used them for library preparation and paired-end sequencing (2 x 250 bp) on an Illumina MiSeq sequencer (Illumina, San Diego, CA, USA) at the Biodesign Institute, Arizona State University, USA.

We processed the Illumina raw reads on the KBase platform using default parameters [3]. Specifically, we trimmed raw reads using Trimmomatic v.0.36 and then *de novo* assembled using metaSPAdes v3.15.3 both with default parameters on the KBase platform[3]. We identified contigs via a BLASTn search of the GenBank database [4]. Subsequently, we mapped the trimmed raw reads to the most similar complete genome from GenBank using the BBMap plugin in Geneious Prime[5] with default parameters. We subsequently performed variant analysis using the ‘find variant’ tool in Geneious Prime with default settings.

To understand how the variant profiles of Ad41 found in WW in this study track with those present in variants publicly available in GenBank, we collected hexon protein gene complete coding sequences of the top 100 variants downloaded from a GenBank search using MW567966 as the query. Precisely, we extracted 59 of the variants from complete genomes while the remaining 41 were not part of complete genomes but had the complete hexon protein coding sequence publicly available in GenBank. We performed multiple sequence alignment (MSA) of Ad41 genomes recovered from GenBank using ClustalW in MEGA X [6], and constructed maximum-likelihood trees using 1,000 bootstrap replicates in IQ-Tree [7]. Prior to phylogenetic tree construction, we used ModelFinder [8] to identify the best-fitting nucleotide substitution model. We used heatmapper [9] to estimate pairwise identity.

**Table S1:** Polymerase chain reaction (PCR) conditions for assays used in this study. Note that the second round (nested) assay was done using GoTaq green PCR master mix. Assays 1 and 2 were done using Phusion green master mix. Please see Table S2 for details of primer sequence.

|  | **Region amplified** | **Amplicon size (bp)** | **Pre-heat** | **# of cycles** | **Denaturation** | **Annealing** | **Extension** | **Final incubation** | **Reference** |
| --- | --- | --- | --- | --- | --- | --- | --- | --- | --- |
| Assay 1 | Complete Genome pool 1 | ~5000 for each of 4 non-overlapping amplicons | 94^o^C – 3 min | 40x | 94^o^C – 30 sec | 55^o^C – 30 sec | 68^o^C – 6 min | 68^o^C – 10 min | This study |
| Assay 2 | Complete Genome pool 2 | ~5000 for each of 4 non-overlapping amplicons | 94^o^C – 3 min | 40x | 94^o^C – 30 sec | 55^o^C – 30 sec | 68^o^C – 6 min | 68^o^C – 10 min | This study |
| Assay 3 | Nested Partial long fiber | ~600 | 95^o^C – 3 min | 35x | 95^o^C – 30 sec | 55^o^C – 30 sec | 60^o^C – 45 sec | 72^o^C – 5 min | [10] |

**Table S2:** Primers used in this study. Primers shown are from 5’ – 3’. Please see Table S1 for details of reaction conditions and source of primers.

| Assay 1 | Ad41-F1 | GCGAGTAGAGTTTTCTCCTCCG |
| --- | --- | --- |
|  | Ad41-R1 | TCAAATCGGTATACGGCGACAC |
|  | Ad41-F3 | TCTTGCATGAGTCTTTCCACGG |
|  | Ad41-R3 | GGGCGAAAAGTTACTGGGTCTT |
|  | Ad41-F5 | GACCCACGATGTAACCACAGAC |
|  | Ad41-R5 | CGGGGACTGCAAAAACTGGATA |
|  | Ad41-F7 | AGCGCTTGGATTACATGAAGATCT |
|  | Ad41-R7 | ACGTTAGTGTTGGCAGACTGTC |
| Assay 2 | Ad41-F2 | GTCTTTCCAAGGCCGAAGAGTT |
|  | Ad41-R2 | TCGTCTTATGCTGGAGGACCTT |
|  | Ad41-F4 | GCTAGAAAGCGACATAGGGGTG |
|  | Ad41-R4 | ACTGTCAACAGCCTGATTCCAC |
|  | Ad41-F6 | TGGGGGTTTGTTATCAGCATGG |
|  | Ad41-R6 | AACCACAGCTGGGTATTTGGAG |
|  | Ad41-F8 | GTCAAGTTTTTCCAGCAGCACC |
|  | Ad41-R8 | GTTCCTCAGCCCCTGATGTTTT |
| Assay 3 | Ad41-Fiber-F | ACTTAATGCTGACACGGGCAC |
|  | Ad41-Fiber-R | TAATGTTTGTGTTACTCCGCTC |

**Table S3:** Summary of Illumina raw reads generated, trimmed and mapped to Ad41 in this study.

|  | **Month-Year** | **Conc-ID** | **Total # Raw reads** | **Total # trimmed reads** | **Total # mapped reads** | **Total # mapped reads (%)** |
| --- | --- | --- | --- | --- | --- | --- |
| Filtrate | Oct-19 | 1 | 1,949,546 | 1,939,546 | 1,016,632 | 52.42 |
| Filtrate | Nov-19 | 2 | 2,275,718 | 2,264,586 | 1,258,868 | 55.59 |
| Filtrate | Dec-19 | 3 | 2,090,046 | 2,080,078 | 1,461,773 | 70.27 |
| Filtrate | Jan-20 | 4 | 2,229,900 | 2,218,484 | 1,128,286 | 50.86 |
| Filtrate | Feb-20 | 5 | 1,997,038 | 1,982,070 | 993,865 | 50.14 |
| Filtrate | Mar-20 | 6 | 2,176,360 | 2,162,018 | 735,266 | 34.01 |
| FTS | Oct-19 | 13 | 2,235,250 | 2,222,254 | 1,307,496 | 58.84 |
| FTS | Nov-19 | 14 | 2,403,492 | 2,392,238 | 1,261,209 | 52.72 |
| FTS | Dec-19 | 15 | 2,430,600 | 2,417,524 | 1,608,144 | 66.52 |
| FTS | Jan-20 | 16 | 2,494,270 | 2,478,854 | 1,128,793 | 45.54 |
| FTS | Feb-20 | 17 | 2,039,716 | 2,029,004 | 1,297,690 | 63.96 |
| FTS | Mar-20 | 18 | 1,869,524 | 1,853,254 | 363,461 | 19.61 |
|  |  | **Total** | **26,191,460** | **26,039,910** | **13,561,483** | **52.08** |

**Table S4:** Amino acid substitutions and deletions detected by variant analysis of mapped reads in long-fiber. Codon numbering is relative to long fiber gene of MW567966 (L1). Note that L1 has the same sequence as the reference. Hence, will have no substitutions and is consequently not listed in this table.

| **S/N** | **Sub** | **codon#** | **L2** | **L3** |
| --- | --- | --- | --- | --- |
| 1 | F – V | 251 | **+** |  |
| 2 | 15aa deletion | 263-277 |  | **+** |

**Table S5:** Long fiber gene variant profile of WW concentrates from October 2019 to March 2020.

| **Month-Year** | **FTS** | **Filtrate** |
| --- | --- | --- |
| Oct-19 | L1 | L1 + L2 |
| Nov-19 | L1 + L2 | L1 + L2 |
| Dec-19 | L1 | L1 |
| Jan-20 | L1 + L3 | L1 |
| Feb-20 | L1 | L1 |
| Mar-20 | L1 | L1 |


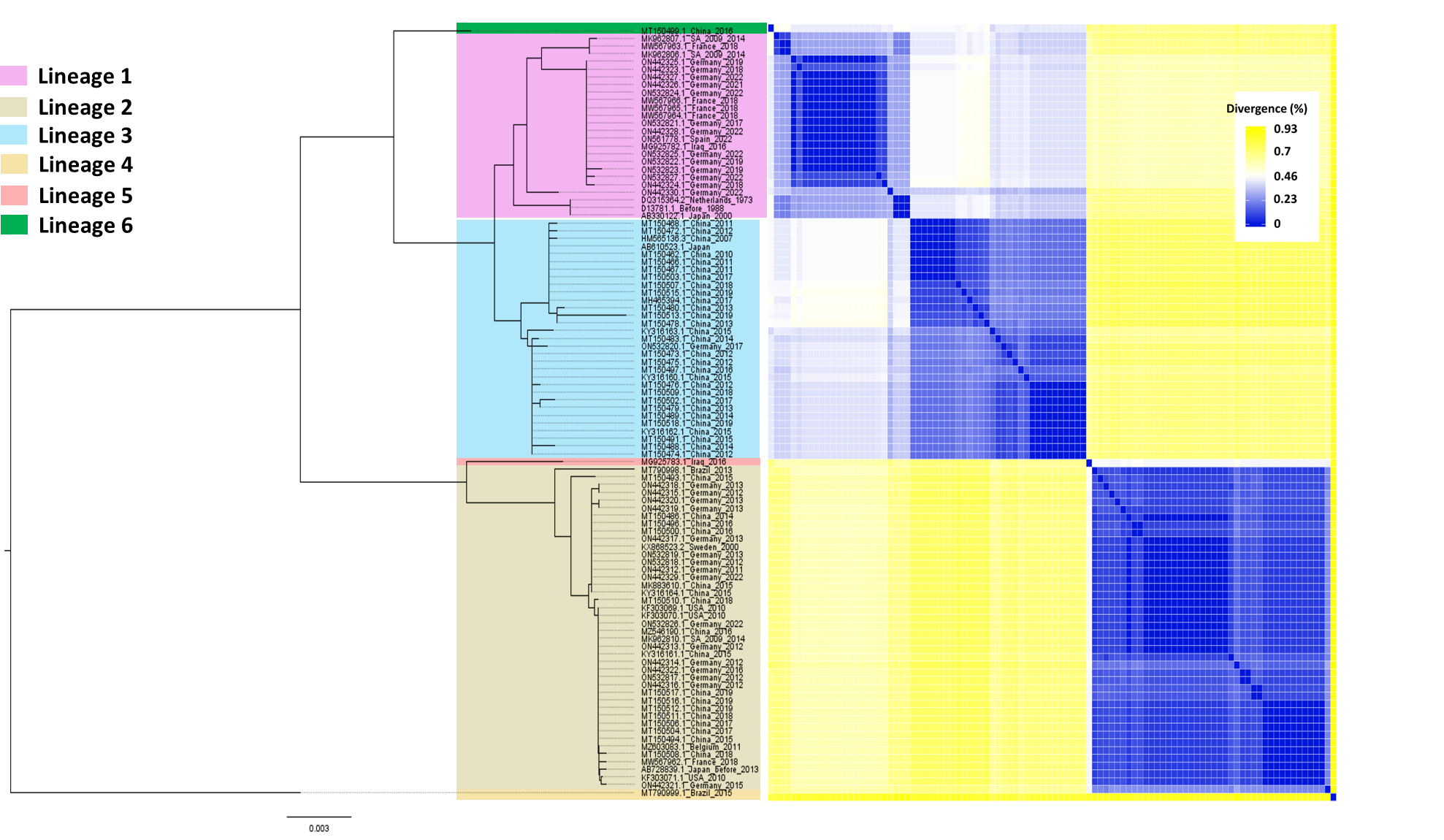


**Figure S1:** Genetic characterization and similarity of Ad41 Hexon protein gene variants. Maximum-likelihood tree and pairwise divergence analysis of Ad41 top 100 hits of a BLASTn search of the GenBank database using MW567966 as the query sequence.


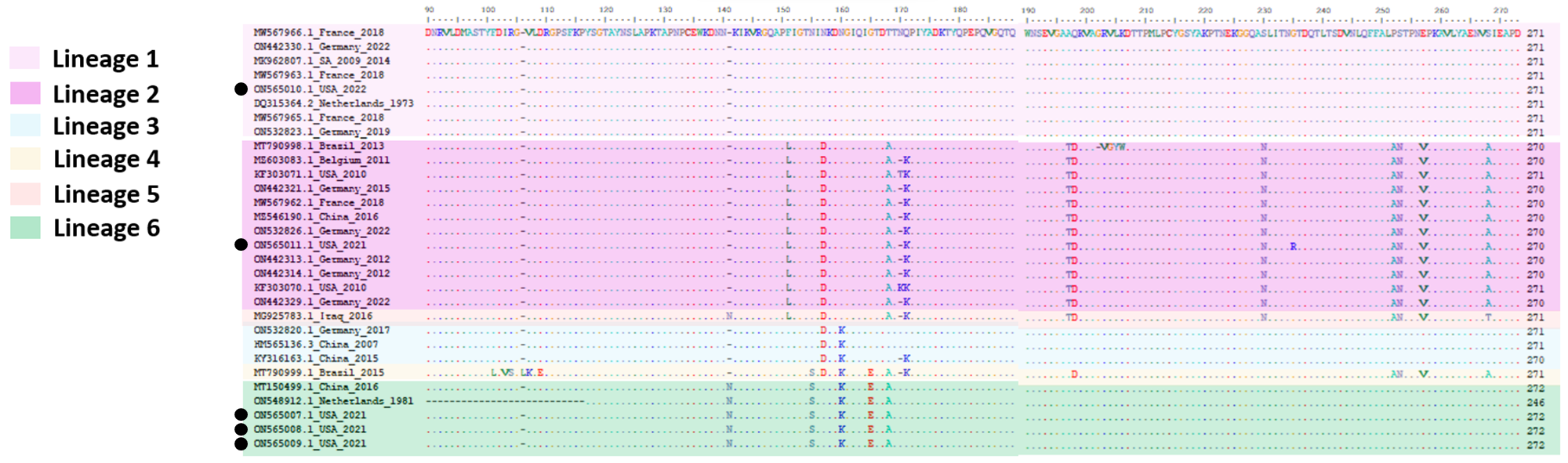


**Figure S2:** A subset of the unique amino acid substitution signatures of Ad41 hexon protein gene protein sequence from representatives of lineages 1-6 (residues 90 – 271; MW567966 numbering, and publicly available sequences of variants recovered from children with hepatitis-of-unknown origin [11] (highlighted with black circles).


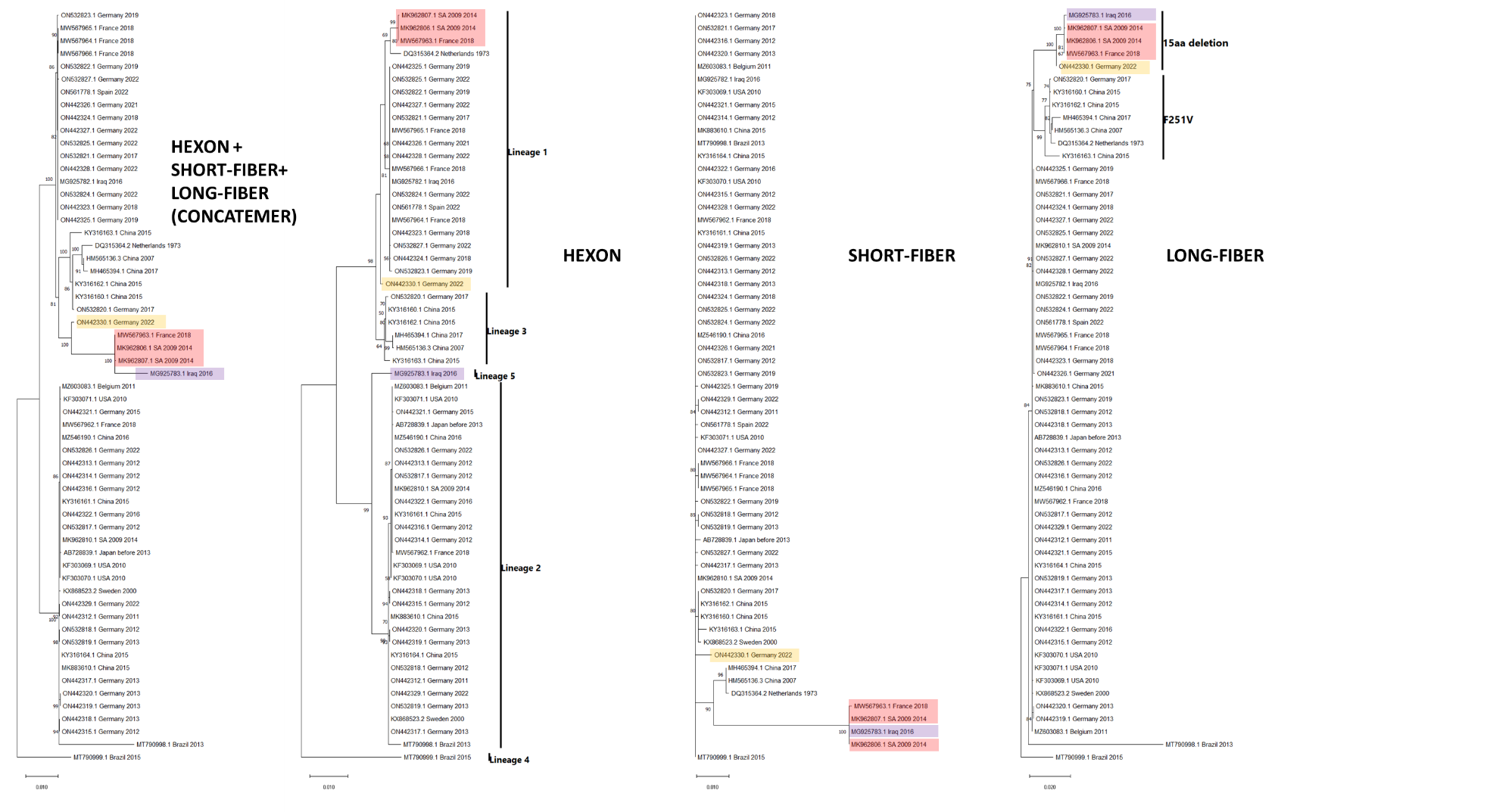


**Figure S3:** Maximum-likelihood tree of Ad 41 variants. The first tree on the left was inferred using concatenated hexon protein, small fiber protein and long fiber protein coding gene sequences extracted from 59 Ad41 complete genomes present in GenBank. The tree was inferred using IQ-Tree 1.6.12. with the best substitution model selected for each of the partitions using ModelFinder. The remaining trees are of hexon (K3P+I substitution model), small fiber (HKY+F substitution model) and long fiber (HKY+F substitution model) protein coding gene sequences extracted from 59 Ad41 complete genomes present in GenBank. These trees were also inferred using IQ-Tree 1.6.12. with the best substitution model selected using ModelFinder. Bootstrap values are shown if greater than 50%. Sequences of interest are color-coded to highlight phylogeny violations between the different trees.


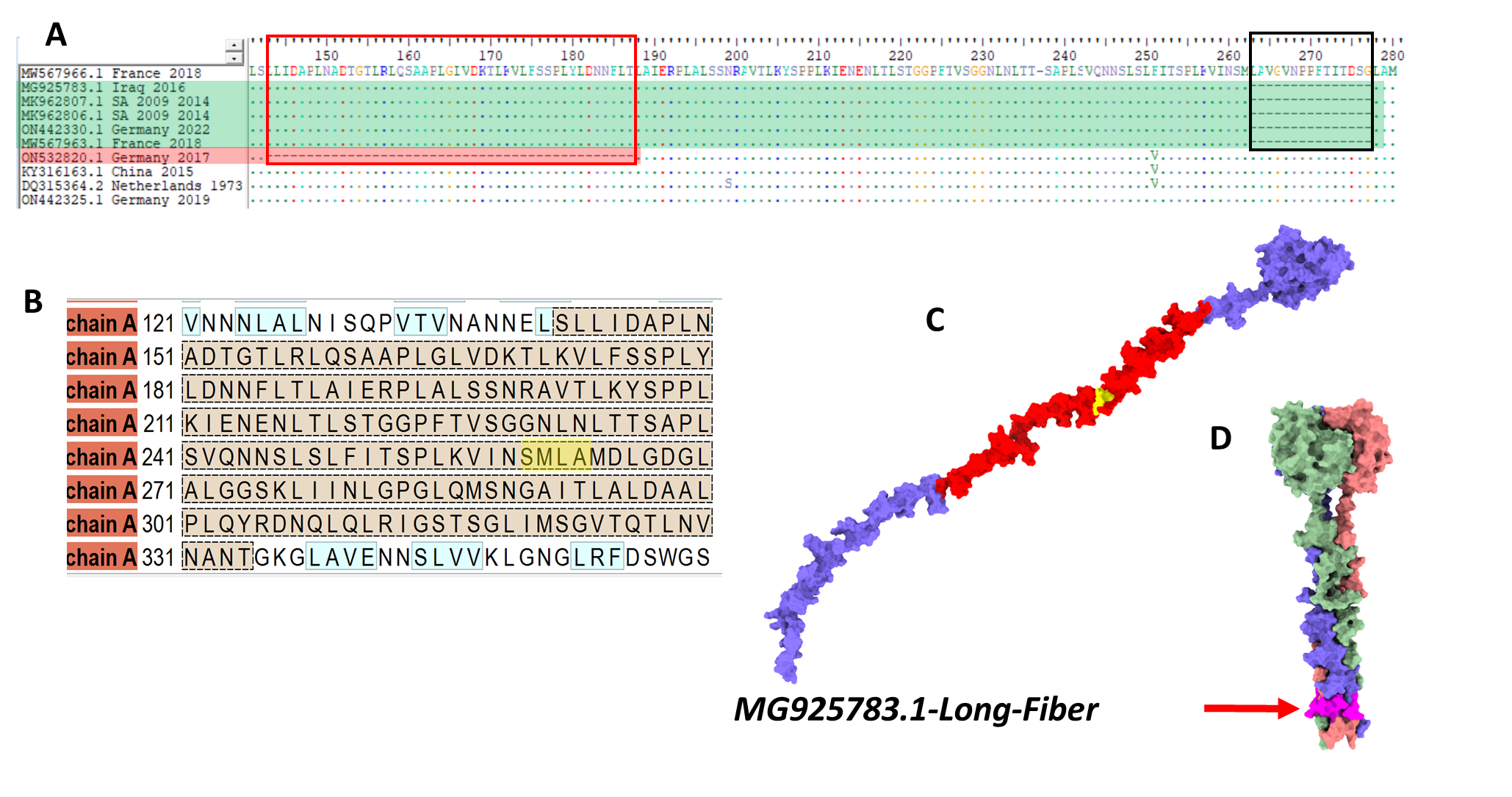


**Figure S4:** Amino acid (aa) analysis of the long fiber of Ad41. A) alignment of a subset of sequences (amino acid 141-280) described in the phylogenetic trees (Figure A3). MG925783, MK962807, MK962808, MW567963 and ON442330 are highlighted in green. Please note the 15aa deletion (LAVGVNPPFTITDSG [black rectangular highlight]) flanked on the N and C termini by SM and LA, respectively. Also note the 45aa deletion (red rectangular highlight) in ON532820 (red highlight). B) A portion of the aa sequence of MG925783 highlighting in brown and yellow the region amplified by the 2^nd^ PCR assay and amino acids SM and LA (flanking the 15 aa deletion), respectively. Note that the first 45aa of the region amplified by the Ad41 2^nd^ round PCR corresponds to the 45aa deletion in ON532820 suggesting that the assay will miss sequences with this deletion. C) ColabFold predicted structure of MG925783 long fiber monomer. Highlighted in red and yellow are the region amplified by the 2^nd^ round PCR assay and the aa (SM and LA) flanking the 15aa deletion, respectively. D) ColabFold predicted trimeric structure of the c-terminal 200aa of long fiber consensus sequence recovered from October 2019 in this study. The portion of the long fiber shaft corresponding to the 15aa deletion is highlighted in pink and pointed to using a red arrow. Structural modelling of Ad41 was done using ColabFold which implements Alphafold2 using MMseqs2 [12]. Predicted structures were viewed and annotated using ChimeraX 1.4. [13].

**References**

1. Faleye TOC, et al. (2022) Impact of sample clarification by size exclusion on virus detection and diversity in wastewater-based epidemiology. *medRxiv : the preprint server for health sciences*, 2022.09.25.22280344. https://doi.org/10.1101/2022.09.25.22280344.

2. Quick J, et al. (2017) Multiplex PCR method for MinION and Illumina sequencing of Zika and other virus genomes directly from clinical samples. *Nature protocols,* 12(6), 1261–1276. https://doi.org/10.1038/nprot.2017.066.

3. Arkin AP, et al. (2018) KBase: The United States Department of Energy Systems Biology Knowledgebase. *Nature biotechnology,* 36(7), 566–569. https://doi.org/10.1038/nbt.4163.

4. Sayers EW, et al. (2021) GenBank. *Nucleic acids research*, 49(D1), D92–D96. https://doi.org/10.1093/nar/gkaa1023.

5. Kearse M, et al, (2012) Geneious Basic: an integrated and extendable desktop software platform for the organization and analysis of sequence data. *Bioinformatics (Oxford, England),* 28(12), 1647–1649. https://doi.org/10.1093/bioinformatics/bts199.

6. Kumar S, et al. (2018) MEGA X: Molecular Evolutionary Genetics Analysis across Computing Platforms. *Molecular biology and evolution,* 35(6), 1547–1549. https://doi.org/10.1093/molbev/msy096.

7. Nguyen LT, et al. (2015) IQ-TREE: a fast and effective stochastic algorithm for estimating maximum-likelihood phylogenies. *Molecular biology and evolution,* 32(1), 268–274. https://doi.org/10.1093/molbev/msu300

8. Kalyaanamoorthy S, et al. (2017) ModelFinder: fast model selection for accurate phylogenetic estimates. *Nature methods,* 14(6), 587–589. https://doi.org/10.1038/nmeth.4285.

9. Babicki S, et al. (2016) Heatmapper: web-enabled heat mapping for all. *Nucleic acids research,* 44(W1), W147–W153. https://doi.org/10.1093/nar/gkw419.

10. Xu, W., et al. (2000) Species-specific identification of human adenoviruses by a multiplex PCR assay. *Journal of clinical microbiology*, 38(11), 4114–4120. https://doi.org/10.1128/JCM.38.11.4114-4120.2000.

11. Gutierrez Sanchez LH, et al. (2022) A Case Series of Children with Acute Hepatitis and Human Adenovirus Infection. *The New England journal of medicine*, 387(7), 620–630. https://doi.org/10.1056/NEJMoa2206294

12. Mirdita M, et al. (2022) ColabFold: making protein folding accessible to all. *Nature methods,* 19(6), 679–682. https://doi.org/10.1038/s41592-022-01488-1.

13. Pettersen EF, et al. (2021) UCSF ChimeraX: Structure visualization for researchers, educators, and developers. *Protein science : a publication of the Protein Society, 30(1), 70–82. https://doi.org/10.1002/pro.3943*.
